# Effect of low doses of biocides on the antimicrobial resistance and the biofilms of *Cronobacter sakazakii* and *Yersinia enterocolitica*

**DOI:** 10.1038/s41598-019-51907-1

**Published:** 2019-11-04

**Authors:** Rosa Capita, María Vicente-Velasco, Cristina Rodríguez-Melcón, Camino García-Fernández, Javier Carballo, Carlos Alonso-Calleja

**Affiliations:** 1 Department of Food Hygiene and Technology, Veterinary Faculty, University of León, E-24071 León, Spain; 2 Institute of Food Science and Technology, University of León, E-24071 León, Spain; 3 Area of Food Technology, University of Vigo, E-32004 Ourense, Spain

**Keywords:** Antimicrobial resistance, Biofilms

## Abstract

The susceptibility of *Cronobacter sakazakii* ATCC 29544 (CS) and *Yersinia enterocolitica* ATCC 9610 (YE) to sodium hypochlorite (10% of active chlorine; SHY), peracetic acid (39% solution of peracetic acid in acetic acid; PAA) and benzalkonium chloride (BZK) was tested. Minimum inhibitory concentration (MIC) values (planktonic cells; microdilution broth method) of 3,800 ppm (SHY), 1,200 ppm (PAA) and 15 ppm (BZK) for CS, and 2,500 ppm (SHY), 1,275 ppm (PAA) and 20 ppm (BZK) for YE, were found. In some instances, an increase in growth rate was observed in presence of sub-MICs (0.25MIC, 0.50MIC or 0.75MIC) of biocides relative to the samples without biocides. The cultures exhibited an acquired tolerance to biocides and an increase in antibiotic resistance after exposure to sub-MICs of such disinfectants. Strains were able to form strong biofilms on polystyrene after 48 hours (confocal laser scanning microscopy), with average biovolumes in the observation field (14,161 µm^2^) of 242,201.0 ± 86,570.9 µm^3^ (CS) and 190,184.5 ± 40,860.3 µm^3^ (YE). Treatment of biofilms for 10 minutes with disinfectants at 1MIC or 2MIC reduced the biovolume of live cells. PAA (YE) and BZK (CS and YE) at 1MIC did not alter the percentage of dead cells relative to non-exposed biofilms, and their effect of countering biofilm was due principally to the detachment of cells. These results suggest that doses of PAA and BZK close to MICs might lead to the dissemination of live bacteria from biofilms with consequent hazards for public health.

## Introduction

*Cronobacter sakazakii* is a Gram-negative, facultative anaerobic bacterium that is mobile and non-spore-forming. It is seen as an emerging opportunist pathogen transmitted in food. Outbreaks of human illness caused by this enterobacterium have been associated with milk formulas for suckling infants, but the microorganism is also linked to a wide range of ready-to-eat foods, such as fresh vegetables and fruits, milk, cheese, meat and fish^1^. The widespread presence of this bacterium permits the contamination of foodstuffs and its ability to grown at low temperatures allows its survival and multiplication under refrigeration^2,3^. Infections by *C. sakazakii* have been detected most often in the elderly and in immunocompromised adults. However, the seriousness of infections is worse in the new-born, where the clinical symptoms of the infection include necrotizing enterocolitis, bacteriaemia and meningitis, with fatality rates between 40% and 80%^4^.

*Yersinia enterocolitica* is the aetiological agent of yersiniosis, the third most frequent zoonotic disease in the European Union (after campylobacteriosis and salmonellosis), with 6,823 cases of human infection confirmed in 2017^5^. Symptoms range from slight, self-limiting gastroenteritis through to acute enteritis with diarrhoea and abdominal pain, mesenteric lymphadenitis and syndromes mimicking appendicitis, particularly in children under five years of age; there may also be septicaemia in the elderly and the immunocompromised^6^. Yersiniosis in humans is due to the ingestion of contaminated foodstuffs, with pigs being considered a key reservoir, because of the high prevalence of virulent strains in these animals^7^. Pigs can be carriers of the microorganism without showing any symptoms, with it being present in the lymphatic glands, the tonsils, the intestinal tract or all of these. During the slaughtering process the bacterium can be spread to different parts of the carcass^8^. Refrigeration is not completely efficacious in controlling this microorganism, since *Y*. *enterocolitica* is a psychrophilic bacterium^9^.

Resistance to antibiotics constitutes a problem of the first magnitude and of growing importance worldwide. The difficulty of treating infections by resistant bacteria involves substantial extra costs for health services, besides having a major impact in terms of morbidity and mortality^10^. It has been suggested that there is a direct linkage between tolerance to biocides and antibiotic resistance, since the mechanisms contributing to both are similar, for instance, changes in cell permeability or the synthesis of efflux pumps^11–13^. On these lines, prior studies performed on enterobacteria (*Escherichia coli* and *Salmonella enterica*) have made it plain that repeated exposure to sub-inhibitory doses of disinfectants triggers an adaptation of bacterial populations to biocides, while also modifying the pattern of resistance to antibiotics in strains^12,14^.

Biofilms are heterogeneous groupings of microorganisms sticking to a surface, included within a polymer matrix secreted by the microorganisms themselves and made up principally of polysaccharides, proteins and nucleic acids^15,16^. This matrix acts as a physical barrier against chemical agents and provides a protective ecological niche enhancing the survival of the microorganisms. This fact, together with the physiological changes undergone by sessile cells, leads to the creation of an environment favourable to the inhabitants of the biofilm, which are protected against various environmental stresses such as drying out or antimicrobials^17–19^. Additionally, because of the difficulty of eliminating them, biofilms raise important problems for the food industry, as they constitute a reservoir of microorganisms, both pathogenic and spoilage, increasing the risk of contamination of foodstuffs in processing plants, with the health and financial repercussions that consequently emerge^20^. In previous research it has been observed that exposure of *E. coli*, *S. enterica*, methicillin-resistant *Staphylococcus aureus* (MRSA) or *Listeria monocytogenes* to sub-inhibitory doses of biocides enhances the capacity of these bacteria to form biofilm^12,17,18,21^.

In the food industry, chemical products are routinely used to disinfect surfaces that are in contact with foodstuffs. Among such products there are sodium hypochlorite (SHY), peracetic acid (PAA) and benzalkonium chloride (BZK). SHY and PAA are approved for use in the European Economic Area (EEA) and Switzerland for various uses, including disinfection of food and feed areas (product-type 4)^22,23^. According to Commission Implementing Regulation (EU) 2017/1273, active chlorine released from SHY is approved as an active substance for use in biocidal product-types 1 (human hygiene biocidal products), 2 (private area and public health disinfectants), 3 (veterinary hygiene biocida products), 4 (food and feed area disinfectants) and 5 (drinking water disinfectants) when active chlorine concentration in aqueous solution is ≤18% w/w (i.e., ≤180,000 ppm). PAA is approved as an active substance for use in biocidal products for product-types 1, 2, 3, 4, 5 and 6 (in-can preservatives, non-food), subject to the specifications and conditions set out in Commission Implementing Regulation (EU) 2016/672. BZK is being reviewed for use in several biocidal product-types, including food and feed area disinfectants, in the EEA area and Switzerland^24^.

There are a number of circumstances in which biocides may be applied in sub-inhibitory doses, for example as a consequence of incorrect calculations of concentrations to be used, of inappropriate storage of the substances leading to a loss of effectiveness, of the presence of excessive amounts of organic material able to inactivate various chlorine compounds like sodium hypochlorite, of an uneven spreading of the biocides or of the difficulty of ensuring disinfectants reach certain areas^12,17,18^. Estimates of microbial growth under different conditions (for instance, in presence of various concentrations of disinfectants) are important when undertaking quantitative evaluation of risks^25^. In studying how microorganisms grow, mathematical models can be used, permitting estimation of various parameters (such as lag phase or maximum growth rate) that describe this growth^26^.

The aim of this study was to evaluate the susceptibility profile of planktonic and sessile cells of *C. sakazakii* and *Y. enterocolitica* to various concentrations of SHY, PAA and BZK. It was also investigated whether contact with sub-inhibitory concentrations of these substances can reduce the susceptibility of the strains to biocides and antibiotics.

## Results and Discussion

### Minimum inhibitory concentrations (MICs) and adaptation

The values for the MICs of SHY, PAA and BZK for *C. sakazakii* ATCC 29544 (CS) and *Y. enterocolitica* ATCC 9610 (YE) are shown in Table 1. Maximum concentrations of biocides that allowed microbial growth after several passages through gradually increasing concentrations of the compounds are also shown.

**Table 1:** Minimum inhibitory concentration (MIC) and maximum concentration of biocides allowing microbial growth after adaptation for *Cronobacter sakazakii* ATCC 29544 and *Yersinia enterocolitica* ATCC 9610.

Bacteria	Biocide	MIC (ppm)^a^	Adaptation (ppm)^b^
*Cronobacter sakazakii*	SHY^c^	3,800	4,275
	PAA^d^	1,200	2,025
	BZK^e^	15	56.95
*Yersinia enterocolitica*	SHY	2,500	6,328.13
	PAA	1,275	2,151.57
	BZK	20	50.63

^a^Minimum inhibitory concentration; ^b^Maximum concentration of biocides that allowed microbial growth after several passages through gradually increasing concentrations of the compounds; ^c^Sodium hypochlorite; ^d^Peracetic acid; ^e^Benzalkonium chloride.

SHY was the substance that required the highest concentrations to inhibit the growth of both strains after 48 hours of incubation (3,800 ppm, equating to 380 ppm of active chlorine, for CS and 2,500 ppm, equivalent to 250 ppm of active chlorine, for YE). The MICs noted for SHY fell within the range previously observed for other enterobacteria, which ran from 390 ppm^14^ to 6,000 ppm^17^. The MIC values for PAA observed in the present study (1,200 ppm for CS and 1,275 ppm for YE) were higher than the MICs of peroxyacids recorded in earlier work for *S. enterica*, at 70 ppm to 80 ppm, and for *L. monocytogenes*, at 100 ppm to 110 ppm^11^. The differences in the results emerging from various research works may be due to the fact that not all microorganisms present the same susceptibility to different biocides. The different compositions of peroxyacid mixtures can also be responsible for the different results found between reports^27^. BZK was the disinfectant that produced inhibition of growth at the lowest concentrations (15 ppm for CS and 20 ppm for YE). These figures are similar to those noted previously with other species of bacteria: 2 ppm (MRSA)^18^, 8 ppm (*S. enterica* serotype Typhimurium)^17^ or 3 ppm to 13 ppm (*L. monocytogenes*)^21^.

After several passes through increasing sub-inhibitory concentrations of biocides, the maximum concentration of SHY that allowed growth of *Y. enterocolitica* was 6,328.13 ppm (a figure 2,53 times higher than the MIC of the strain not exposed in this way), as shown in Table 1. However, in the case of *C. sakazakii* such a marked adaptation was not achieved for SHY. Both strains presented a similar behaviour when affected by PAA, since they showed similar MIC values, and in both instances the maximum concentration at which the strains grew after exposure to sub-inhibitory doses of the biocides was double the level of the MIC noted before this exposure. Adaptation to BZK was also striking, especially in the case of *C. sakazakii*, where the maximum concentration permitting microbial growth after exposure to sub-inhibitory doses of biocides was 3.8 times higher than the MIC for this strain prior to exposure. The reduction in susceptibility to biocides after repeated exposure of the strains to sub-inhibitory amounts of these substances has also been noted in previous studies of other enterobacteria, such as *E. coli*^12^ or *S. enterica*^11,14,17^.

In food-processing environments, biocides are sometimes used at sub-inhibitory doses^12,28^. The reduction in susceptibility to biocides after they are applicated in small amounts may be due to advantageous chromosomal mutations triggering modifications in cells, principally in the composition and structure of the external membrane, preventing penetration by antimicrobial agents. This resistance may also be associated with phenotypic modifications, as a consequence of metabolic regulation of responses to the stresses induced by the presence of biocides (e.g., expression of efflux mechanisms)^10^. In any case, so as to reduce the risk of increasing tolerance to biocides, lethal concentrations of these substances should be used, whether in a clinical or in a food-processing context.

### Growth curves

**Figure 1:**
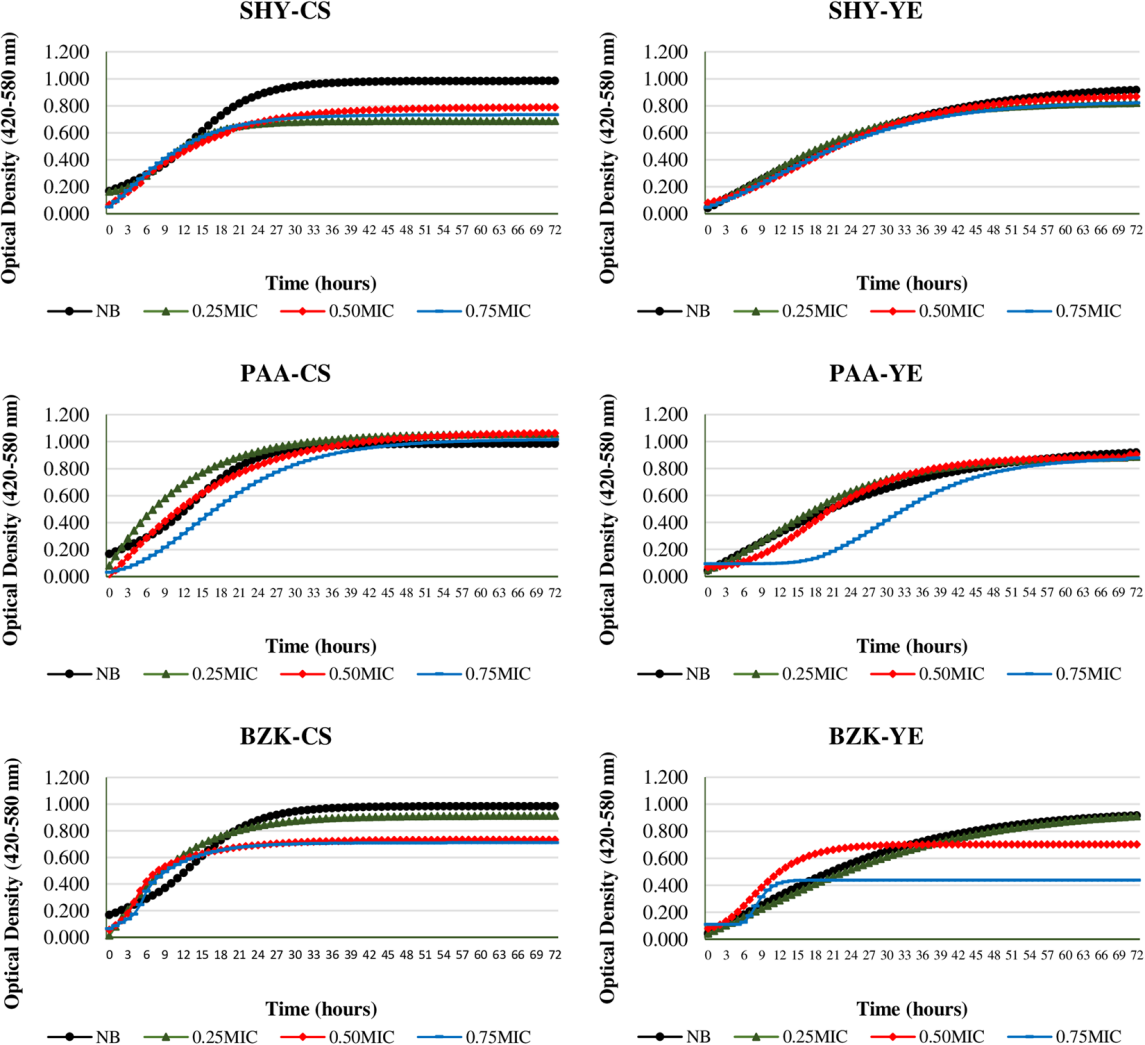
Growth curves of *Cronobacter sakazakii* ATCC 29544 (CS) and *Yersinia enterocolitica* ATCC 9610 (YE) in tryptone soy broth without biocides (NB) or with various concentrations of sodium hypochlorite (SHY), peracetic acid (PAA) or benzalkonium chloride (BZK). Minimum inhibitory concentration (MIC) values for SHY, PAA and BZK were 3,800 ppm, 1,200 ppm and 15 ppm, respectively, in the case of CS, and 2,500 ppm, 1,275 ppm and 20 ppm, respectively, for YE.

Including the replicates, a total of 60 growth curves were generated for the strains tested, determining the optical density at 420 nm to 580 nm (OD_420–580_), and then fitting the data to the modified Gompertz equation. The comparative growth curves in presence of different types (SHY, PAA and BZK) and concentrations (0.25MIC, 0.50MIC and 0.75MIC) of disinfectants are shown in Fig. 1. Tables 2 and 3 show the estimated growth kinetic parameters (lag phase, maximum growth rate and maximum OD_420–580_ at the stationary phase). The R^2^ values for the Gompertz model fit were high (>0.90).

**Table 2:** Lag phase (L; hours), maximum growth rate (µ; ΔOD_420–580_/h) and maximum OD_420–580_ in the stationary phase (D) for *Cronobacter sakazakii* ATCC 29544 in the presence of various biocides.

Biocide	Concentration of biocide	Growth kinetic parameter		
		L	µ	D
Without biocide		5.840 ± 2.706 f	0.055 ± 0.005 ab	0.982 ± 0.086 a
Sodium hypochlorite	**0.25MIC**	2.840 ± 0.400 ef	0.040 ± 0.010 a	0.690 ± 0.130 a
	**0.50MIC**	−6.167 ± 2.255 cd	0.032 ± 0.004 a	0.788 ± 0.025 a
	**0.75MIC**	−4.100 ± 2.052 d	0.044 ± 0.010 a	0.783 ± 0.163 a
Peracetic acid	**0.25MIC**	−24.333 ± 5.132 a	0.123 ± 0.025 d	0.960 ± 0.609 a
	**0.50MIC**	−9.973 ± 1.435 bc	0.042 ± 0.010 a	1.191 ± 0.251 a
	**0.75MIC**	3.219 ± 0.119 ef	0.031 ± 0.004 a	0.700 ± 0.569 a
Benzalkonium chloride	**0.25MIC**	−8.000 ± 1.900 bcd	0.069 ± 0.020 abc	0.936 ± 0.047 a
	**0.50MIC**	−11.000 ± 2.000 b	0.102 ± 0.023 cd	0.626 ± 0.247 a
	**0.75MIC**	0.407 ± 1.810 e	0.093 ± 0.057 bcd	0.707 ± 0.147 a

Minimum inhibitory concentration (MIC) values were 3,800 ppm, 1,200 ppm and 15 ppm for sodium hypochlorite, peracetic acid and benzalkonium chloride, respectively. Data (mean ± SD; n = 3) in the same column with no letters in common are significantly different (*P* < 0.05).

**Table 3:** Lag phase (L; hours), maximum growth rate (µ; ΔOD_420–580_/h) and maximum OD_420–580_ in the stationary phase (D) for *Yersinia enterocolitica* ATCC 9610 in the presence of various biocides.

Biocide	Concentration of biocide	Growth kinetic parameter		
		L	µ	D
Without biocide		−4.617 ± 1.119 c	0.022 ± 0.003 a	0.887 ± 0.070 c
Sodium hypochlorite	**0.25MIC**	−4.637 ± 1.554 c	0.023 ± 0.003 a	0.753 ± 0.067 b
	**0.50MIC**	−6.487 ± 0.484 b	0.022 ± 0.002 a	0.770 ± 0.087 b
	**0.75MIC**	−11.410 ± 0.610 a	0.025 ± 0.005 ab	0.767 ± 0.083 b
Peracetic acid	**0.25MIC**	−3.727 ± 1.546 c	0.028 ± 0.002 ab	0.890 ± 0.020 c
	**0.50MIC**	6.963 ± 0,629 e	0.031 ± 0,001 b	0.883 ± 0,040 c
	**0.75MIC**	19.283 ± 0,654 f	0.028 ± 0,002 ab	0.880 ± 0,020 c
Benzalkonium chloride	**0.25MIC**	−12.487 ± 0.480 a	0.021 ± 0.002 a	0.963 ± 0.031 c
	**0.50MIC**	2.170 ± 0.170 d	0.050 ± 0.010 c	0.700 ± 0.080 b
	**0.75MIC**	6.233 ± 0.451 e	0.074 ± 0.004 d	0.443 ± 0.025 a

Minimum inhibitory concentration (MIC) values were 2,500 ppm, 1,275 ppm and 20 ppm for sodium hypochlorite, peracetic acid and benzalkonium chloride, respectively. Data (mean ± SD; n = 3) in the same column with no letters in common are significaltly different (*P* < 0.05).

In some instances (*C. sakazakii* in the presence of 0.25MIC PAA, 0.50MIC BZK and 0.75MIC BZK, and *Y. enterocolitica* exposed to 0.50MIC PAA, 0.50MIC BZK and 0.75MIC BZK) an increased growth rate was observed relative to the samples without biocides. In prior studies^29,30^, higher rates of microbial growth have also been seen in samples with biocides than in the control samples. In the case of *Y. enterocolitica*, the increase in the growth rate was associated with a longer lag phase, with regard to controls. It is suggested that this prolongation of the lag phase could enable the bacteria to adapt to the compound to a certain extent, thus increasing their growth rate when the lag phase ends. In the case of *C. sakazakii*, bacterial populations exposed to 0.25MIC PAA, 0.50MIC BZK or 0.75MIC BZK apparently showed a higher growth rate and a shorter lag phase than controls, a result which is hard to explain and which would imply that in the presence of these substances the strains immediately begin growing at a rapid rate.

In respect of OD_420–580_ in the stationary phase, no significant differences were observed between treated and control samples for *C. sakazakii*. The relatively large standard deviations observed could be responsible for the absence of significant differences. In contrast, where *Y. enterocolitica* was concerned, SHY at the three concentrations tested and BZK at 0.50MIC and 0.75MIC permitted a slight reduction in OD_420–580_ in the stationary phase in relation to the control samples (which had no biocides). The scant differences found between the growth parameters of the samples with and without biocides suggests that the sub-inhibitory concentrations of biocides tested are ineffective as antimicrobial agents and may even encourage bacterial growth, especially with regard to *C. sakazakii* exposed to small doses of PAA and BZK.

### Susceptibility to antibiotics

Infections caused by bacteria resistant to antibiotics are normally hard to treat, since many of the substances habitually employed in clinical practice are ruled out as therapeutic options. Resistance to antibiotics is a problem of growing dimensions, currently considered as one of the greatest challenges facing public health worldwide^10^. In this study it was determined whether exposure of *C. sakazakii* ATCC 29544 and *Y. enterocolitica* ATCC 9610 to small amounts of biocides could contribute to growing resistance to antibiotics. To this end, strains were screened for susceptibility to 15 antibiotics of clinical importance before and after exposure to increasing sub-inhibitory concentrations of biocides. The antibiotic resistance patterns of the strains tested are shown in Table 4.

**Table 4:** Antimicrobial susceptibility pattern of *Cronobacter sakazakii* ATCC 29544 (CS) and *Yersinia enterocolitica* 9610 (YE) before and after exposure to increasing sub-inhibitory concentrations of biocides.

Bacteria	Antibiotic														
	AMP	TE	CIP	C	SXT	NA	AMC	CAZ	IMP	ATM	CTX	FOX	CN	AK	STR
CS no exposed	R	S	S	S	S	S	S	S	S	S	S	S	S	S	S
CS exposed to SHY	S	S	R	S	S	S	S	S	S	S	S	S	S	S	S
CS exposed to PAA	S	S	S	S	S	S	S	S	S	S	S	S	S	S	S
CS exposed to BZK	R	S	I	S	S	S	S	S	S	S	S	S	S	S	S
YE no exposed	R	S	S	S	S	S	S	S	S	S	S	S	S	S	S
YE exposed to SHY	S	S	S	S	S	S	S	S	S	S	R	R	S	S	S
YE exposed to PAA	S	S	S	S	S	S	S	S	S	S	R	R	S	S	I
YE exposed to BZK	R	S	S	S	S	S	S	S	S	S	S	S	S	S	S

AMP, ampicillin (10 µg); TE, tetracycline (30 µg); CIP, ciprofloxacin (5 µg); C, chloramphenicol (30 µg); SXT, trimethoprim/sulfamethoxazole (25 µg); NA, nalidixic acid (30 µg); AMC, amoxycillin/clavulanic acid (30 µg); CAZ, ceftazidime (30 µg); IPM, imipenem (10 µg); ATM, aztreonam (30 µg); CTX, cefotaxime (30 µg); FOX, cefoxitin (30 µg); CN, gentamicin (10 µg); AK, amikacin (30 µg); STR, streptomycin (25 µg). SHY, sodium hypochlorite; PAA, peracetic acid; BZK, benzalkonium chloride. R, resistant strain; I, intermediate strain; S, susceptible strain. The increase in resistance was defined as a change from S (before exposure) to I or R (after exposure) according to the CLSI guidelines.

Prior to exposure, strains showed resistance only to AMP. In a number of research works, it has been made clear that enterobacteria produce α- and β-lactamases which confer resistance to this antibiotic^31–33^. Some researchers have observed that the presence of efflux pumps may also be linked with resistance to AMP^34^.

After exposure to increasing sub-inhibitory concentrations of biocides, the strains of *C. sakazakii* and *Y. enterocolitica* exhibited resistance to various antibiotics, which were not detected before biocide exposition. This happened with ciprofloxacin (CS exposed to SHY), cefotaxime and cefoxitin (YE exposed to SHY and PAA). In other instance the growth in adaptive resistance was not so marked, moving strains from the category of “susceptible” to the category of “intermediate”, as occurred with ciprofloxacin (CS exposed to BZK) and streptomycin (YE exposed to PAA). For various additional cultures and antibiotics, the diameter of inhibition zones in exposed cells decreased with regard to unexposed, but did not exceed the limit of resistance according to the Clinical and Laboratory Standards Institute of the U.S.A. (CLSI) guidelines, and the strains remained within the category of “susceptible”. It has been suggested that even a modest change in susceptibility would still be significant because it might confer a growth advantage on a strain^35^.

Changes in the antibiotic susceptibility patterns of microbial species following exposure to biocides have been reported for various classes of chemical substances, suggesting that tolerance to biocides are associated with a broad-spectrum mechanisms (e.g. efflux pumps or permeability alterations). For instance, the antibiotics CIP, CTX, FOX and STR, to which a reduction in susceptibility were observed after exposure to sub-inhibitory doses of biocides, are known efflux pump substrates^36,37^.

The results of this research support the findings of other authors who have also observed that exposure to increasing sub-lethal concentrations of biocides (including food and feedstuff preservatives, disinfectants or decontaminants) selects for antibiotic resistance in bacteria^14,36,38^. Results in the present study are of note, in view of the fact that CIP, CTX, FOX and STR are classified as “critically important” antimicrobials for human medicine^39^. In the World Organization for Animal Health list^40^, CIP and STR are classified as “veterinary critically important antimicrobials”.

It is a remarkable finding that after the exposure to SHY and PAA, the bacterial strains exhibited susceptibility to AMP. An increase in antibiotic-susceptibility after exposure to biocides has also been reported in previous studies for several antibiotics^14,41^ and has been suggested to be due to a potential increase in cell permeability in response to biocide adaptation. However, the underlying mechanisms remain unclear^42^. It should be noted that an increase in susceptibility to clinically relevant antibiotics in previously resistant pathogenic bacteria would be a beneficial circumstance when biocides are applied in clinical and food environments.

### Biofilms of *C. sakazakii* and *Y. enterocolitica*

**Figure 2:**
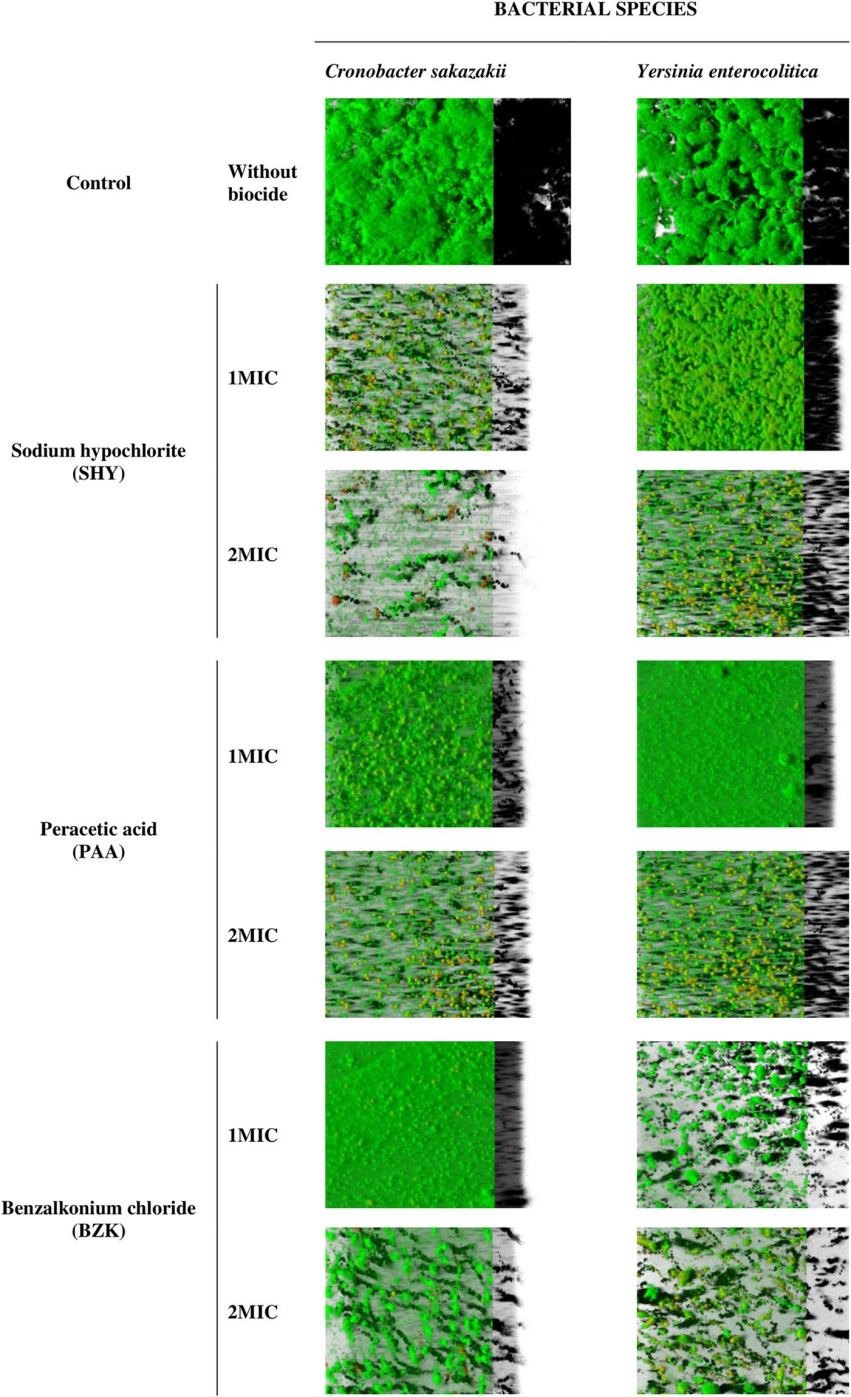
Three-dimensional projections of 48-hour-old biofilm structures of *Cronobacter sakazakii* ATCC 29544 (CS) and *Yersinia enterocolitica* ATCC 9610 (YE) after treatment for ten minutes with sodium hypochlorite (SHY), peracetic acid (PAA) or benzalkonium chloride (BZK) at different concentrations. Images (119 µm × 119 µm) were reconstructed from confocal z-stacks using IMARIS 9.1 software, with the shadow projections on the right. Minimum inhibitory concentration (MIC) values for CS are 3,800 ppm (SHY), 1,200 ppm (PAA) and 15 ppm (BZK). MIC values for YE are 2,500 ppm (SHY), 1,275 ppm (PAA) and 20 ppm (BZK).

Biofilms formed in food-processing environments are a potential source of contamination by pathogenic and spoilage microorganisms. Moreover, these structures enhance the resistance of bacterial cells to various environmental stresses, such as drying out or antimicrobial treatments. Hence, it is of interest to learn the capacity of pathogenic bacteria to form biofilms, as also the resistance of such structures to different biocide chemicals. The structures representative of biofilms formed by *C. sakazakii* ATCC 29544 (CS) and *Y. enterocolitica* 9610 (YE) on polystyrene after 48 hours of incubation are shown in Fig. 2. Tables 5 and 6 show the values for structural parameters of biofilms. When the biofilms grew in the absence of biocides (control), *C. sakazakii* and *Y. enterocolitica* formed compact structures covering most of the surface, with a biovolume in the observational field (14,161 µm^2^) of 242,201.0 ± 86,570.9 µm^3^ for CS and 190,184.5 ± 40,860.3 µm^3^ for YE. The percentage of surface cover was 99.69 ± 0.35 µm for CS and 96.62 ± 1.91 µm for YE. This biovolume is greater than that previously observed for other enterobacteria, such as *E. coli* ATCC 12806 (23,664.6 ± 2,703.9 µm^3^)^12^, *S. enterica* serotype Typhimurium (S175; 129,358.3 ± 34,659.3 µm^3^)^17^ or *S. enterica* serotype Hadar (SH174; 73,073.6 ± 52,365.9 μm^3^)^19^. Other researchers have also demonstrated the considerable capacity of strains of *C. sakazakii* and *Y. enterocolitica* to form biofilms, whether on inert surfaces^43,44^ or on organic surfaces, for example, cucumbers in the case of *C. sakazakii*^45^ and pig meat in that of *Y. enterocolitica*^46^.

**Table 5:** Structural parameter values for the 48-hour-old biofilms formed on polystyrene by *Cronobacter sakazakii* ATCC 29544 after treatment for ten minutes with sodium hypochlorite (SHY), peracetic acid (PAA) or benzalkonium chloride (BZK) at different concentrations.

Biocide	Total biovolume (µm^3^)	Biovolume of life cells (µm^3^)	Biovolume of dead cells (µm^3^)	% Surface coverage	Maximum thickness (µm)	Average thickness (µm)	Roughness
Control	242,201.0 ± 86,570.9 c	241,422.2 ± 86,486.9 c	778.8 ± 205.5 a	99.69 ± 0.35 d	42.00 ± 7.00 b	17.09 ± 6.12 c	0.361 ± 0.067 a
SHY-1MIC	31,140.5 ± 1,796.1 ab	28,135.3 ± 1,633.7 ab	3,005.3 ± 394.9 a	65.72 ± 1.79 b	17.67 ± 0.58 a	2.04 ± 0.08 ab	0.926 ± 0.051 b
SHY-2MIC	18,249.4 ± 9,128.9 a	15,182.8 ± 6,434.0 a	3,066.6 ± 2,698.2 a	34.62 ± 3.25 a	24.67 ± 4.93 a	1.29 ± 0.64 a	1.194 ± 0.250 c
PAA-1MIC	81,470.5 ± 8,075.9 ab	73,294.9 ± 5,060.7 ab	8,175.6 ± 3,175.1 c	99.71 ± 0.12 d	22.00 ± 2.65 a	5.75 ± 0.57 ab	0.345 ± 0.029 a
PAA-2MIC	36,720.8 ± 5,636.6 ab	29,596.2 ± 2,287.0 ab	7,124.7 ± 3,417.0 bc	76.21 ± 2.36 c	19.00 ± 4.58 a	2.59 ± 0.40 ab	0.798 ± 0.027 b
BZK-1MIC	91,387.6 ± 5,346.1 b	90,536.4 ± 4,526.8 b	517.7 ± 419.8 a	99.99 ± 0.01 d	23.00 ± 1.00 a	6.43 ± 0.34 b	0.180 ± 0.010 a
BZK-2MIC	35,594.4 ± 9,789.9 ab	31,525.0 ± 9,416.4 ab	4,069.5 ± 584.7 ab	74.71 ± 8.80 c	24.00 ± 2.65 a	2.47 ± 0.41 ab	0.841 ± 0.091 b

Data (mean ± SD; n = 9) in the same column with no letters in common are significantly different (*P* < 0.05). Minimum inhibitory concentration (MIC) values for *C. sakazakii* ATCC 29544 are 3,800 ppm (SHY), 1,200 ppm (PAA) and 15 ppm (BZK).

**Table 6:** Structural parameter values for the 48-hour-old biofilms formed on polystyrene by *Yersinia enterocolitica* ATCC 9610 after treatment for ten minutes with sodium hypochlorite (SHY), peracetic acid (PAA) or benzalkonium chloride (BZK) at different concentrations.

Biocide	Total biovolume (µm^3^)	Biovolume of life cells (µm^3^)	Biovolume of dead cells (µm^3^)	% Surface coverage	Maximum thickness (µm)	Average thickness (µm)	Roughness
Control	190,184.5 ± 40,860.3 c	189,517.2 ± 40,906.8 c	667.4 ± 195.7 a	96.62 ± 1.91 c	37.00 ± 2.65 e	13.43 ± 2.88 c	0.462 ± 0.073 bc
SHY-1MIC	124,444.7 ± 9,079.5 b	108,983.8 ± 1,216.6 b	15,460.9 ± 3,120.2 c	99.59 ± 0.17 c	21.67 ± 2.08 c	8.79 ± 0.64 b	0.287 ± 0.017 ab
SHY-2MIC	38,345.0 ± 4,854.8 a	28,969.5 ± 4,775.5 a	9,375.5 ± 640.2 b	86.95 ± 6.69 b	26.67 ± 4.93 d	2.71 ± 0.34 a	0.678 ± 0.158 c
PAA-1MIC	99,745.4 ± 4,688.6 b	99,510.6 ± 4,714.7 b	234.4 ± 27.8 a	99.99 ± 0.01 c	16.00 ± 1.00 ab	7.05 ± 0.33 b	0.150 ± 0.008 a
PAA-2MIC	32,717.0 ± 6,929.4 a	31,171.6 ± 6,890.3 a	1,545.4 ± 139.1 a	70.06 ± 5.43 a	17.67 ± 0.58 bc	2.31 ± 0.49 a	0.529 ± 0.071 c
BZK-1MIC	17,930.4 ± 2,489.2 a	17,888.0 ± 2,189.1 a	42.3 ± 7.8 a	64.79 ± 3.79 a	11.67 ± 2.52 a	1.10 ± 0.09 a	1.160 ± 0.085 e
BZK-2MIC	18,129.9 ± 5,908.0 a	17,469.8 ± 5,913.5 a	660.7 ± 92.1 a	70.65 ± 10.38 a	19.00 ± 3.46 bc	1.28 ± 0.42 a	0.924 ± 0.269 d

Data (mean ± SD; n = 9) in the same column with no letters in common are significaltly different (*P* < 0.05). Minimum inhibitory concentration (MIC) values for *Y. enterocolitica* ATCC 9610 are 2,500 ppm (SHY), 1,275 ppm (PAA) and 20 ppm (BZK).

The effects of exposure for ten minutes to 1MIC and 2MIC of sodium hypochlorite (SHY), peracetic acid (PAA) and benzalkonium chloride (BZK) on structural parameters of biofilms were determined. All the treatments tested were effective in reducing the biovolume of *C. sakazakii* and *Y. enterocolitica* relative to control samples. This was more marked for the highest concentrations of disinfectants, even though major differences were observed as a function of treatment and strain. After exposure to SHY at 1MIC, the biovolume of biofilms decreased (*P* < 0.05) relative to control biofilms both in *C. sakazakii* (biovolume of 31,140.5 ± 1,796.1 µm^3^) and in *Y. enterocolitica* (124,444.7 ± 9,079.5 µm^3^). No more than a few groupings of isolated cells were seen after exposure to SHY at 2MIC (Fig. 2); the biovolume was 18,249.4 ± 9,128.9 µm^3^ for *C. sakazakii* and 38,345.0 ± 4,854.8 µm^3^ for *Y. enterocolitica*.

After treatment with PAA at 1MIC, and compared to untreated control biofilms, a decrease in the biovolume was observed in both *C. sakazakii* (81,470.5 ± 8,075.9 µm^3^) and *Y. enterocolitica* (99,745.4 ± 4,688.6 µm^3^) biofilms. These changes can be related to reduction in biofilm thickness, since the percentage of surface covered by sessile cells remained close to 100%. Treatment with PAA at 2MIC substantially reduced the biovolume, percentage of surface covered and thickness of biofilms, in a similar way for both microorganisms. This may be explicable in terms of the broad spectrum of action of this biocide^47^. With regard to the antimicrobial efficacy of PAA on sessile cells, varying results have been published. Certain authors observed that PAA at 250 ppm was less effective than SHY at 100 ppm in reducing biofilms formed by *E. coli* on the equipment of a poultry-processing plant^48^. In contrast, other researchers determined that PAA was the most effective disinfectant against biofilms, as compared with other disinfectants in common use that they tested^49,50^.

Finally, treatment with BZK produced a different effect on the two bacterial species. In the case of CS the biovolume of the biofilm was 91,387.6 ± 5,346.1 µm^3^ after treatment with 1MIC of BZK and 35,594.4 ± 9,789.9 µm^3^ after the use of 2MIC. BZK caused intense shedding of cells from biofilms of *Y. enterocolitica*, both at 1MIC and at 2MIC, with biovolumes of 17,930.4 ± 2,489.2 µm^3^ and 18,129.9 ± 5,908.0 µm^3^, respectively. The reduction in the biovolume of biofilms formed by *L. monocytogenes* after treatment with BZK at concentrations of 0.5MIC, 1MIC or 1.5MIC has been observed in previous reports^51^.

**Figure 3:**
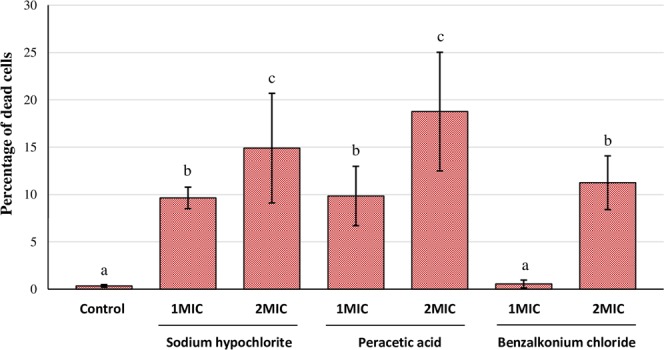
Percentage of dead cells relative to total cells in biofilms formed by 48-hour-old biofilms of *Cronobacter sakazakii* ATCC 29544 (CS) after treatment for ten minutes with sodium hypochlorite (SHY), peracetic acid (PAA) or benzalkonium chloride (BZK) at different concentrations. Bars (mean ± SD; n = 9) with no letters in common are significantly different (*P* < 0.05). Minimum inhibitory concentration (MIC) values for CS are 3,800 ppm (SHY), 1,200 ppm (PAA) and 15 ppm (BZK).

**Figure 4:**
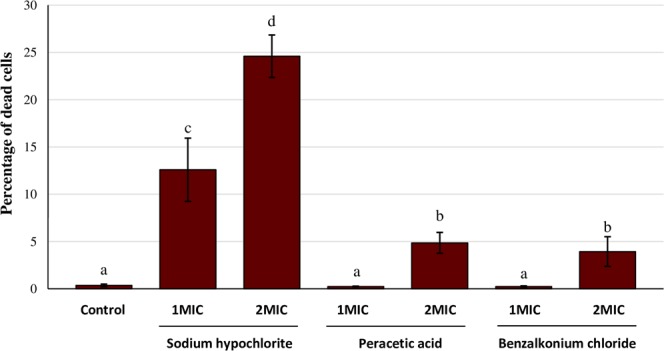
Percentage of dead cells relative to total cells in biofilms formed by 48-hour-old biofilms of *Yersinia enterocolitica* ATCC 9610 (YE) after treatment for ten minutes with sodium hypochlorite (SHY), peracetic acid (PAA) or benzalkonium chloride (BZK) at different concentrations. Bars (mean ± SD; n = 9) with no letters in common are significantly different (*P* < 0.05). Minimum inhibitory concentration (MIC) values for CS are 2,500 ppm (SHY), 1,275 ppm (PAA) and 20 ppm (BZK).

The percentage of dead bacteria (calculated as the biovolume of dead bacteria with respect to total biovolume) in control biofilms (untreated) was 0.342 ± 0.138% (*C. sakazakii*; Fig. 3) and 0.363 ± 0.135% (*Y. enterocolitica*; Fig. 4). The percentage of dead cells increased (*P* <0.05) after treatments with biocides, with the exception of the use of PAA at 1MIC (*Y. enterocolitica*) and of BZK at 1MIC (*C. sakazakii* and *Y. enterocolitica*), in which percentages of inactivated cells were similar (*P* > 0.05) to those in control biofilms.

In view of the scant loss of viability after treatment with PAA or BZK at 1MIC, it may be surmised that the anti-biofilm effect of these treatments is linked principally with the shedding of cells (considerable in the case of *Y. enterocolitica*). These results suggest that the use of these biocides at concentrations close to MICs may cause the dissemination of live bacteria from the biofilm, involving a potential risk for food safety. Similar results have previously been noted in respect of *L. monocytogenes*^21^.

The highest percentages of dead cells were observed after treatment with PAA at 2MIC when *C. sakazakii* was concerned (18.764% ± 6.265%) and with SHY at 2MIC for *Y. enterocolitica* (24.605% ± 2.251%). These results are coincident with those emerging from a study undertaken with MRSA, in which the percentage of dead cells was lower when BZK was used than when SHY was utilized^18^.

It is concluded that exposure to increasing sub-inhibitory concentrations of SHY, PAA or BZK triggered adaptive tolerance of these compounds in strains of *C. sakazakii* ATCC 29544 and *Y. enterocolitica* ATCC 9610. These strains also evinced a reduced susceptibility to certain antibiotics of medical importance (ciprofloxacin, cefotaxime, cefoxitin and streptomycin). These two facts underline the crucial need to avoid the application of low (close to MIC) concentrations of biocides in food-processing environments. Moreover, study of microbial populations over time in the presence of sub-MICs of biocides demonstrated the inability of these compounds to make any substantial impact on the growth parameters of the bacteria with regard to control samples (without biocides). Both *C. sakazakii* and *Y. enterocolitica* were capable of producing robust biofilms on polystyrene, a worrying fact when it is kept in mind that this plastic is frequently used on various surfaces and in a range of equipment in the food industry. When were used at 1MIC, PAA (*Y. enterocolitica*) and BZK (*C. sakazakii* and *Y. enterocolitica*), produced only a scant inactivation of cells, while causing a marked shedding of cells from the biofilm. This could contribute to the dissemination of live cells, with a consequent risk for food safety. The present research work expands knowledge of the behaviour of planktonic and sessile cells of *C. sakazakii* and *Y. enterocolitica* when confronted with small amounts of various disinfectants in common use in the food industry. This may assist in ensuring a more effective and safer use of these compounds.

## Methods

### Bacteria and biocides

*C. sakazakii* ATCC 29544 (CS) and *Y. enterocolitica* ATCC 9610 (YE) were tested. The strains were stored at −50 °C in tryptone soya broth (TSB, Oxoid Ltd., Hampshire, United Kingdom) with 20% (vol/vol) of glycerol. During the experiments the cultures were kept at 4 °C on tryptone soya agar plates (TSA, Oxoid).

The biocides studied were sodium hypochlorite (10% of active chlorine; SHY, Sigma-Aldrich Co., St. Louis, Missouri, U.S.A.), peracetic acid (39% solution of peracetic acid in acetic acid; PAA, Sigma-Aldrich) and benzalkonium chloride (BZK, Sigma-Aldrich). Solutions of chemicals were prepared aseptically in sterile distilled water immediately before experiments.

### Determination of minimum inhibitory concentrations (MICs)

Values for MICs were established by means of a method involving microdilution in broth in accordance with the norms of the Clinical and Laboratory Standards Institute of the U.S.A. (CLSI)^52^. Five colonies were taken from the TSA plates and inoculated into tubes with 10 ml of TSB, then incubated at 30 °C. After 24 hours, these bacterial cultures contained approximately 8 log_10_ colony-forming units per millilitre (cfu/ml). The wells of 100-well polystyrene microplates (Oy Growth Curves Ab Ltd., Helsinki, Finland) were filled with 20 microlitres of the biocide solution, use being made of a range of concentrations for each substance, and 180 microlitres of the third dilution of bacterial culture, so as to achieve a concentration of approximately 5 log_10_ cfu/ml in the well. Positive controls were included (200 μl of inoculum), as were negative controls (180 μl of TSB plus 20 μl of the chemical under test). After incubation for 48 hours at 30 °C, optical density at 420 nm to 580 nm (OD_420–580_) was determined using a Bioscreen C MRB (Oy Growth Curves Ab). MIC was established as the lowest concentration of biocide necessary to avoid growth after 48 hours of incubation^12^. On the basis of previous experimentation, the limit of growth was taken to be an OD_420–580_ of 0.200^20^.

### Exposure to increasing sub-inhibitory concentrations of biocides

With the aim of determining any possible adaptation by the strains, they were exposed to increasing sub-inhibitory concentrations of the disinfectants in 100-well polystyrene microplates (Oy Growth Curves Ab). The starting concentration of the biocides was MIC/2. The first well was filled with 180 μl of the third dilution of each of the strains and 20 μl of the biocide solution. When growth was observed in the well, 20 μl of the suspension was transferred aseptically to the next well, to which 160 μl of TSB were added, along with 20 μl of biocide solution. Each well had a concentration of biocide one-and-a-half times greater than the previous well. This process was repeated until no growth was observed after 48 hours of incubation at 30 °C.

The last suspension with growth in the well was inoculated onto TSA plates with biocide (using half of the maximum concentration of biocide allowing microbial growth) which were incubated at 30 °C for 48 hours. Both exposed and unexposed strains were simultaneously tested for antibiotic susceptibility after the same number of days of storage on agar plates.

### Growth curves

In order to draw up growth curves the third dilution of CS and YE (inoculated and incubated as indicated above) was used. To each well of 100-well polystyrene microplates (Oy Growth Curves Ab), 180 μl of the strain and 20 μl of the biocide solution were added (the final concentration of biocide in the well was 0.25MIC, 0.50MIC or 0.75MIC). The optical density OD_420–580_ was determined from hour 0 through to hour 72 at intervals of one hour in a Bioscreen C MBR (Oy Growth Curves Ab). The micro-titre plates were agitated for one minute before turbidity was measured. The model utilized to adjust the growth curves to the data recorded was a modified Gompertz equation^53^: ODt = A + B * exp(−exp(2.71828183 * µ * (L − t)/B + 1)), where t is the time in hours that has elapsed since inoculation, ODt is the optical density (measured in the range 420 nm to 580 nm) at time t, L is the lag time in hours at the end of the lag period, µ is the maximum growth rate achieved (ΔOD_420-580_/h), B is the increase in OD_420–580_ from inoculation to the stationary phase (D), and A is the upper asymptotic curve (OD_420-580_ in the stationary stage, D) minus B.

Values for L, µ and D were obtained for each strain and replication by fitting a sigmoidal curve to the data set using a Marquardt algorithm that calculates those parameter values which give the minimum residual sum of squares. The goodness of fit was evaluated using the coefficient of determination (R^2^). The experiment was repeated in triplicate on different days.

### Tests for antibiotic susceptibility

The strains were investigated to determine their susceptibility before and after exposure disinfectants, on Muller-Hinton agar (MH, Oxoid) using the disc diffusion method. A total of 15 antibiotics (Oxoid) were tested: AMP, ampicillin (10 µg); TE, tetracycline (30 µg); CIP, ciprofloxacin (5 µg); C, chloramphenicol (30 µg); SXT, trimethoprim/sulfamethoxazole (25 µg); NA, nalidixic acid (30 µg); AMC, amoxycillin/clavulanic acid (30 µg); CAZ, ceftazidime (30 µg); IPM, imipenem (10 µg); ATM, aztreonam (30 µg); CTX, cefotaxime (30 µg); FOX, cefoxitin (30 µg); CN, gentamicin (10 µg); AK, amikacin (30 µg); STR, streptomycin (25 µg). The discs were placed on the MH plates which had previously been inoculated with the strains and were incubated for 24 hours at 30 °C. After incubation, inhibition zones were measured and the strains were classified as susceptible, intermediate (with reduced susceptibility) or resistant on the basis of CLSI criteria^54^.

### Study of the biofilms

The structure of the biofilms was investigated using a method described in a previous publication^17^, with slight modifications. The strains were inoculated in ten-millilitre tubes of TSB and incubated at 30 °C for 24 hours, after which two dilutions in the same culture broth were made to obtain a concentration of approximately 6 log_10_ cfu/ml. Quantities of 250 μl were added to the wells of polystyrene micro-titre plates (Matrix 96-Well Polystyrene Flat Bottom microplates; Thermo Fisher Scientific, New Hampshire, U.S.A.), which were incubated for one hour at 30 °C to allow fixation to the wells by the bacteria. Once this time had elapsed, the wells were washed with sodium chloride 150 mM to eliminate non-adherent cells, and 250 μl of sterile TSB were added. The plates were incubated for 48 hours at 30 °C. After incubation, the wells were washed twice with sodium chloride 150 mM, and filled up with sterile distilled water as a control or with solutions of the disinfectants (prepared with sterile distilled water) at 1MIC or 2MIC. After 10 minutes of contact, the wells were emptied and staining was undertaken. A volume of 2.0 µl of a 1:1 mixture of SYTO9 (stock 3.34 mM in DMSO) and propidium iodide (PI; stock 20 mM in DMSO) fluorescent dyes from the BacLight Viability Kit (Invitrogen, Carlsbad, California, U.S.A.) was added to 1,000 µl of TSB, and 250 µl of this solution was added to each well. The plate was then incubated in the dark at 30 °C for 20 minutes to enable fluorescent labelling of the bacteria.

To view the biofilms a Nikon Eclipse TE 2000-U confocal scanning laser microscope (CLSM) was used with the EZ-C13.60 program (Nikon Instruments Inc., New York, U.S.A.). All the biofilms were scanned at 400 Hz, with a forty magnifications lens being used as the objective element. Fluorescence was detected through excitation at 488 nm with an argon laser and emissions were captured with a filter for 590/50 (SYTO9) or 650LP (propidium iodide). During CLSM imaging, SYTO9 emits green fluorescence and is used to identify living microorganisms with an intact membrane whereas PI emits red fluorescence and stains dead bacteria with a damaged membrane. For each biofilm formed, three images were obtained (512 pixels × 512 pixels, corresponding to 119 μm × 119 μm) in three zones of each well selected at random. All these determinations were repeated on three different days. For image processing the program IMARIS 9.1 (Bitplane, Zurich, Switzerland) was used. The structural parameters of the biofilms (biovolume, percentage of surface covered, thickness and roughness) were obtained by using the method described in previous papers^12,55^.

### Statistical analysis

The kinetic growth parameters (L, μ and D) and the structural parameters of the biofilms were analysed using analysis of variance (ANOVA) techniques. Differences between measurements were obtained by means of Duncan’s multiple range test. Significant differences were established for a probability level of 5% (*P* <0.05). Processing of all the data was performed using the computer program Statistica® 8.0 (StatSoft Ltd., Tulsa, Oklahoma, U.S.A.).
